# Structured Kernel Subspace Learning for Autonomous Robot Navigation †

**DOI:** 10.3390/s18020582

**Published:** 2018-02-14

**Authors:** Eunwoo Kim, Sungjoon Choi, Songhwai Oh

**Affiliations:** Department of Electrical and Computer Engineering and ASRI, Seoul National University, Seoul 08826, Korea; kewoo15@snu.ac.kr (E.K.); sungjoon.choi@cpslab.snu.ac.kr (S.C.)

**Keywords:** kernel subspace learning, low-rank approximation, Gaussian processes, motion prediction, motion control

## Abstract

This paper considers two important problems for autonomous robot navigation in a dynamic environment, where the goal is to predict pedestrian motion and control a robot with the prediction for safe navigation. While there are several methods for predicting the motion of a pedestrian and controlling a robot to avoid incoming pedestrians, it is still difficult to safely navigate in a dynamic environment due to challenges, such as the varying quality and complexity of training data with unwanted noises. This paper addresses these challenges simultaneously by proposing a robust kernel subspace learning algorithm based on the recent advances in nuclear-norm and $l_{1}$-norm minimization. We model the motion of a pedestrian and the robot controller using Gaussian processes. The proposed method efficiently approximates a kernel matrix used in Gaussian process regression by learning low-rank structured matrix (with symmetric positive semi-definiteness) to find an orthogonal basis, which eliminates the effects of erroneous and inconsistent data. Based on structured kernel subspace learning, we propose a robust motion model and motion controller for safe navigation in dynamic environments. We evaluate the proposed robust kernel learning in various tasks, including regression, motion prediction, and motion control problems, and demonstrate that the proposed learning-based systems are robust against outliers and outperform existing regression and navigation methods.

## 1. Introduction

In real-world environments, it is difficult for service robots to adapt and assist humans due to complex and crowded situations [1]. Because of the dynamic operating environment, service robots can easily collide with humans, leading to dangerous situations. It is normally required for service robots to predict motions of humans and moving objects and control safely without any collisions for successful navigation. Therefore, we focus on safe navigation of a mobile robot under human–robot coexisting dynamic environments in this paper. There are two significant issues when operating a robot in such environments: predicting dynamic behaviors of pedestrians and finding corresponding controls of a robot.

Autonomous robot navigation has been studied extensively in recent years [2,3,4,5,6,7,8,9,10,11,12,13,14,15,16]. In their studies, the future trajectories of moving humans and objects are estimated for collision-free safe navigation of a robot. In [2], future motion of humans or moving obstacles is modeled into a probabilistic framework of sequential decision problem, which integrates the localization and collision avoidance. In [4], an inverse reinforcement learning method using maximum entropy is proposed to address partial observation to mimic human behavior. Asaula et al. [5] presented a stochastic modeling of human behavior to reduce collision with obstacles by predicting the probability of dangerous situation in human–robot coexisting environments. Fulgenzi et al. [3] utilized a Gaussian process (GP) to model motion patterns of pedestrians. Lam et al. [7] proposed a practical navigation strategy based on harmonious rules and a sensitive region with a safety guarantee in a human–robot shared environment. Jetchev and Toussaint [10] proposed a speed up trajectory prediction approach based on sparse regularized feature selection and efficient trajectory transfer. Many approaches assume the availability of the current positions of moving objects including a robot and obstacles [4,8,12] or predictability of positions from infrastructure, such as an environment with an overhead camera network [5,11]. However, it is impractical to use external structures in many real environments since they are expensive and available only in a laboratory setting.

Recently, Choi et al. [13] proposed to model complex motion patterns of dynamic obstacles based on an autoregressive Gaussian process (AR-GP) and developed a motion controller for safe navigation of a robot. AR-GP is cancapture dynamic human behavior by utilizing a nonlinear, nonparametric regression technique, called Gaussian process regression [17]. The AR-GP based method does not require external devices to collect location information due to its data-driven and egocentric properties. In [13], the authors have shown that the presented method performs better than existing reactive control methods for motion control problems, such as the reactive planner [9] and vector field histogram [18]. Note that the limitation of their work is to collect noise-free training set to perform well, which is a nontrivial task ignoring natural noises in sensors.

To handle outliers in an estimation problem, $l_{1}$-norm based approaches are widely used to robustly solve problems in the presence of outliers [19,20,21]. These techniques are used to represent a robust low-dimensional subspace of the original data in many fields [19,22]. Kim et al. [23] proposed a new robust navigation system for a mobile robot by extending the work in [13], where they approximate a target matrix containing noises and outliers as a low-rank kernel matrix associated with the robust $l_{1}$-norm to remove the undesirable effects derived from measurement noises in the training set. While their system shows the robustness against outliers, it can lead to an infeasible solution due to the lack of the positive semi-definiteness property of the target kernel matrix, making an unstable and even a dangerous situation when a robot based on the system navigates under human–robot coexisting environments. Hence, it is necessary to satisfy the underlying property of a kernel matrix for safe and guaranteed situations.

In this paper, we propose a novel factorization-based Gaussian process regression method, called FactGP, based on structured kernel subspace learning for motion prediction and motion control problems. The proposed motion prediction algorithm, FactGP${}_{M}$, assumes that a kernel matrix with noises can be approximated by a few representative factors while producing a robust solution. By extracting orthonormal basis vectors from a nuclear-norm regularized $l_{1}$-norm minimization problem satisfying symmetric positive semi-definiteness of the solution matrix, we can also reduce the computational complexity since the need for inverting a kernel matrix is no longer required. We also propose a robust motion controller, FactGP${}_{C}$, using the low-rank optimization technique to reduce the effects of unwanted or inconsistent control examples. The proposed structure kernel subspace learning is applied to an extensive set of regression problems including motion prediction in simulation under the existence of noises to demonstrate its robustness. Moreover, it is applied to various motion control experiments to verify its performance. Finally, we conducted experiments in physical environments using a Pioneer 3DX mobile robot with Microsoft Kinect cameras to demonstrate the excellence of the proposed method with respect to safe navigation and robust regression under crowded and dynamic scenarios.

A preliminary version of this work appeared in [24]. The current work extends [24] and introduces an efficient motion controller using structured low-rank optimization. In addition, an extensive set of simulations and experiments for controlling a robot in dynamic environments is included in the current work.

The remainder of this paper is organized as follows: In Section 2 and Section 3, we propose a robust kernel subspace learning algorithm using structured low-rank matrix approximation and describe FactGP${}_{M}$, a motion prediction algorithm. The motion control problem is discussed and FactGP${}_{C}$ is proposed in Section 4. We present various experimental results including real-world experiments to evaluate the proposed method in Section 5.

## 2. Kernel Subspace Learning

The kernel subspace learning refers to approximating a target kernel matrix efficiently using a small number of dominant factors, and in this work we try to solve the next position or control of a robot given recent positions of moving obstacles using autoregressive Gaussian process regression where a kernel matrix leaning is involved. In this section, we present the basic concept of our proposal based on low-rank kernel approximation by analyzing the kernel matrix in Gaussian process regression (GPR).

For GPR, it is required to compute the inversion of a kernel matrix, which usually takes high computational cost. To handle such issue, many approximation methods have been proposed to reduce the heavy complexity of computing an inverse kernel matrix, such as incomplete Cholesky factorization [25] and the Nyström approximation [26]. Following this, we consider a factorization strategy of a kernel matrix with the concept of low-rank-ness, which involves the $l_{2}$-norm function that can address Gaussian noises. Exploiting factorized principal components giving a low-dimensional structure is known as kernel principal component analysis (KPCA) [27]. More specifically, principal components of a kernel matrix by KPCA are exploited by performing linear operations of standard PCA in a high-dimensional feature space [27]. By the kernel approximation using KPCA, we can reduce the computational cost in computing kernel matrix related tasks to speed up kernel machine.

Let $\Phi :R^{n_{x}}\to X$ be a nonlinear mapping from the original input space to a feature space. Then, the covariance matrix is computed for centered data $x_{1},\ldots ,x_{n}$ as

$$C=\frac{1}{n}\sum_{i=1}^{n}\Phi \left(x_{i}\right)\Phi {\left(x_{i}\right)}^{T}$$

and an eigenvector v associated with a nonzero eigenvalue of *C* is $v=\sum_{i=1}^{n}\beta_{i}\Phi \left(x_{i}\right)$. The coefficients $\beta ={\left[\beta_{1}\cdots \beta_{n}\right]}^{T}$ are computed using the following problem [27]:

(1) Kβ=nλβ,

where *K* is a kernel matrix such that ${\left[K\right]}_{ij}=\langle \Phi \left(x_{i}\right),\Phi \left(x_{j}\right)\rangle$. Here, principal components in X can be obtained using top *r* largest eigenvectors, $v_{k}$ for k=1,…,r, over the entire eigenvectors of *K* using their corresponding eigenvalues which are computed with a proper normalization based on coefficients from Equation (1). Hence, a kernel matrix can be represented by a few dominant eigenvectors which correspond to *r* largest eigenvalues.

**Figure 1:**
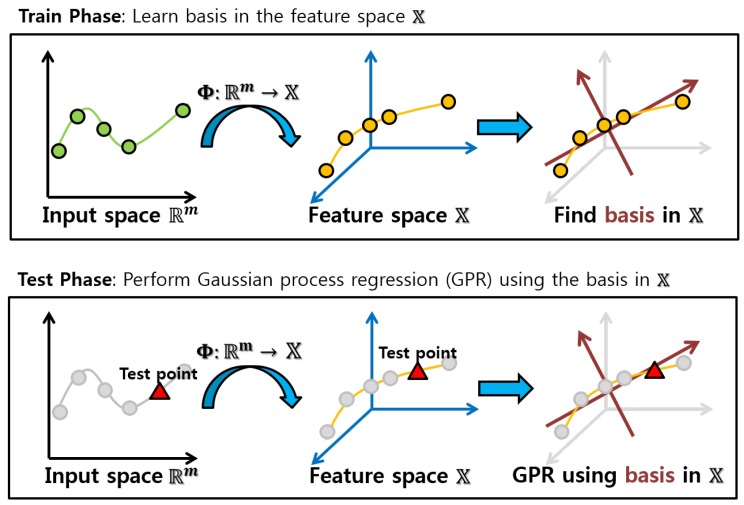
A conceptual illustration of the kernel subspace learning in Gaussian process regression (GPR) [23] (reproduced with permission from Eunwoo Kim, Sungjoon Choi, Songhwai Oh, A Robust Autoregressive Gaussian Process Motion Model Using *l*_1_-Norm Based Low-Rank Kernel Matrix Approximation; published by IEEE 2014), where we perform GPR in the low-dimensional feature space.

Now, we can approximate the inverse of a kernel matrix *K* based on the eigenvalue decomposition:

(2) $$K^{-1}={\left(R\Sigma R^{T}\right)}^{-1}=R\Sigma^{-1}R^{T}\approx \tilde{R}{\tilde{R}}^{T},$$

where $\tilde{R}=R_{r}\Sigma_{r}^{-\frac{1}{2}}$. Here, $R_{r}\in R^{n\times r}$ represents the first *r* vectors from *R* and $\Sigma_{r}=\mathrm{diag}\left(\lambda_{1},\cdots ,\lambda_{r}\right)\in R^{r\times r}$ is a diagonal matrix of *r* largest eigenvalues such that $\lambda_{1}\geq \cdots \geq \lambda_{n}$. Let us define the conditional distribution in Gaussian process regression [17] for a new output $y_{*}$ at a new input $x_{*}$ given $D=\{\left(x_{i},y_{i}\right)|i=1,\ldots ,n\}$

(3) $$y_{*}|D,x_{*}\;∼\;N\left({\bar{y}}_{*},\sigma_{y_{*}}^{2}\right),$$

where

(4) $${\bar{y}}_{*}=k_{*}^{T}{\left(\overset{˘}{K}+\sigma_{w}^{2}I\right)}^{-1}y=k_{*}^{T}K^{-1}y,$$

and $\overset{˘}{K}$ is a kernel matrix such that ${\left[\overset{˘}{K}\right]}_{ij}=k\left(x_{i},x_{j}\right)$. We can combine Equation (2) with Equation (4) as

(5) $${\bar{y}}_{*}=k_{*}^{T}K^{-1}y\approx k_{*}^{T}\tilde{R}{\tilde{R}}^{T}y={\tilde{k}}_{*}^{T}\tilde{y},$$

where ${\tilde{k}}_{*}^{T}=k_{*}^{T}\tilde{R}$ is a projected kernel vector into the orthogonal feature space by the projection matrix $\tilde{R}$ and $\tilde{y}={\tilde{R}}^{T}y$ is a projected output by $\tilde{R}$. This means that ${\tilde{k}}_{*}$ and $\tilde{y}$ act as new representative factors by the orthogonal projection for Gaussian process regression problems, with conversion of the inverse of a kernel matrix into an identity matrix which reveals independence among basis vectors. Hence, Equation (5) is a new representation over ${\bar{y}}_{*}$ in the low-dimensional orthogonal feature space. A conceptual representation of the kernel subspace learning with the low-rank property used in Gaussian process regression is illustrated in Figure 1.

In addition, *K* can be approximated by a conventional low-rank matrix factorization method which transforms data into a low-dimensional subspace using the $l_{2}$-norm. However, the $l_{2}$-norm based method is sensitive to outliers because the $l_{2}$ loss function can amplify the negative effects of the unwanted noises. Therefore, $l_{2}$-norm based low-rank approximation methods may find projections which are far from the desired solution due to the corruptions. As an alternative, various approaches using the $l_{1}$-norm have been proposed recently and it is known that $l_{1}$-norm based methods find a sparse solution, which are more robust against outliers [19,20,21]. Recently, Kim et al. [23] approximated a kernel matrix based on the $l_{1}$-norm for robust regression:

(6) $$\min_{U,V}\;J\left(U,V\right)={∥K-UV∥}_{1},$$

where $K\in R^{n\times n}$, $U\in R^{n\times r}$, and $V\in R^{r\times n}$ are the kernel, projection, and coefficient matrices, respectively. Here, we want to find a low-rank representation UV of *K* with sparse approximation errors, such that the effects of outliers can be reduced. However, the optimization technique in [23] may not be proper when approximating a kernel matrix since the low-rank representation is a bilinear multiplication and thus may not satisfy the positive semi-definiteness of a kernel matrix.

## 3. Proposed Method: FactGP${}_{M}$

In this section, we first propose a structured kernel subspace learning guaranteed with the positive semi-definiteness property of the approximated target matrix. Then, we describe the overall framework using Gaussian process regression for modeling motion.

### 3.1. Formulation

For robust approximation of erroneous data, we formulate the approximation problem based on the robust $l_{1}$-norm. We also apply the recent advances in rank minimization for an automatic rank search of an uncertain rank structure of a kernel matrix [22] (Note that [22] solves the nuclear-norm based optimization problem by iterative thresholding over singular values obtained from singular value decomposition of a measurement matrix, which leads to the automatic rank search. However, the proposed framework fixes the rank of the target matrix $PMP^{T}$. Nonetheless, it has an effect of reducing the rank of the target matrix further from the pre-determined rank).

The nuclear-norm regularized kernel matrix approximation with the $l_{1}$-norm can be formulated as follows:

(7) $$\min_{P,M}\;∥K-PMP^{T}{∥}_{1}+\lambda {∥PMP^{T}∥}_{*},$$

subject to positive semi-definite matrix *M*, where $K\in R^{n\times n}$ is an observed kernel matrix and $P\in R^{n\times r}$ and $M\in R^{r\times r}$ are optimization variables. The nuclear-norm is denoted as ${∥\cdot ∥}_{*}$ and λ>0 is a small scalar value. In the problem, the nuclear-norm regularizer is used to optimize the rank of $PMP^{T}$, an approximation of *K* since it is difficult to find the exact rank of a kernel matrix for real-world problems. Since the problem is typically non-convex, its solution can be computed under the augmented Lagrangian with guarantees [22].

**Figure 2:**
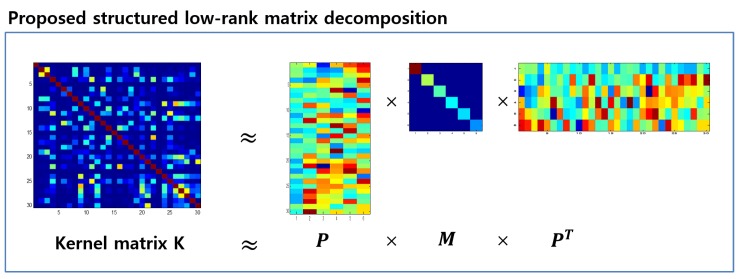
Graphical representation of the kernel matrix factorization [24] (reproduced with permission from Eunwoo Kim, Sungjoon Choi, Songhwai Oh, Structured Low-Rank Matrix Approximation in Gaussian Process Regression for Autonomous Robot Navigation; published by IEEE, 2015), where a kernel matrix *K* is decomposed into three factors. Based on the decomposition, we can learn AR-GP with the low-rank kernel matrix.

Moreover, we constrain an orthogonality property to the basis matrix *P* to reduce the computational cost with faster convergence since the property shrinks the solution space of *P*, which we reformulate the above problem as follows:

(8) $$\begin{array}{cc} \min_{P,M}\;∥K & -PMP^{T}{∥}_{1}+\lambda {∥M∥}_{*}\\ \text{s.t.}\;\; & P^{T}P=I_{r},\;\;M⪰0, \end{array}$$

where $I_{r}$ and *M* are an identity matrix of r×r size and a matrix of positive semi-definite, respectively. Due to the orthogonality constraint on *P*, a small-size matrix *M* is involved in the nuclear-norm function instead of $PMP^{T}$, which expedites solving the problem in Equation (8). The graphical illustration of the structured kernel matrix approximation is described in Figure 2. Since it is difficult to solve the problem in Equation (8) directly, two auxiliary variables, *D* and $\hat{M}$, are introduced to relax the problem as

(9) $$\begin{array}{cc} \min_{P,M,D,\hat{M}} & {∥K-D∥}_{1}+\lambda {∥M∥}_{*}\\ \text{s.t.}\;\;D=P\hat{M}P^{T}, & \;\hat{M}=M,P^{T}P=I_{r},M⪰0. \end{array}$$

To solve for Equation (9), we construct an augmented Lagrangian which handles the constrained optimization using the unconstrained counterpart:

(10) $$\begin{array}{cc} L(K,P,M,D,\hat{M}) & ={∥K-D∥}_{1}+\lambda {∥M∥}_{*}+\mathrm{tr}\left(\Lambda_{1}^{T}\left(D-P\hat{M}P^{T}\right)\right)+\mathrm{tr}\left(\Lambda_{2}^{T}\left(\hat{M}-M\right)\right)\\ & +\frac{\beta }{2}\left(∥D-P\hat{M}P^{T}{∥}_{F}^{2}+{∥\hat{M}-M∥}_{F}^{2}\right), \end{array}$$

subject to the constraints $P^{T}P=I_{r}$ and M⪰0, where $\Lambda_{1},\Lambda_{2}\in R^{n\times n}$ are Lagrange multipliers and β>0 is a parameter to adjust penalty in the optimization problem. We solve Equation (10) using the alternating direction method, which computes one variable while fixing other optimization variables. In the following section, we describe details of each step of the proposed method.

### 3.2. Algorithm

To solve for *M*, we solve the following problem:

(11) $$\begin{array}{cc} M_{+} & =\arg\min_{M}\frac{\lambda }{\beta }{∥M∥}_{*}+\frac{1}{2}{∥\hat{M}-M+\frac{\Lambda_{2}}{\beta }∥}_{F}^{2},\\ & =\arg\min_{M}\frac{\lambda }{\beta }{∥M∥}_{*}+\frac{1}{2}{∥M-A∥}_{F}^{2},\;\text{s.t.}\;M⪰0, \end{array}$$

where $A=\hat{M}-\frac{\Lambda_{2}}{\beta }$. In the case that *A* is asymmetric, we first convert it to a symmetric matrix by $A\leftarrow \frac{A+A^{T}}{2}$ and find $M_{+}$. The solution can be obtained by performing eigenvalue thresholding (EVT) [28]:

(12) $$M_{+}=Q\mathrm{diag}\left[\max\left(\gamma -\frac{\lambda }{\beta },0\right)\right]Q^{T},$$

where *Q* and Γ=diag(γ) are eigenvectors and eigenvalues with compatible size, respectively.

For *D*, we solve the following optimization problem:

(13) $$\begin{array}{cc} D_{+} & =\arg\min_{D}{∥K-D∥}_{1}+\mathrm{tr}\left(\Lambda_{1}^{T}\left(D-P\hat{M}P^{T}\right)\right)+\frac{\beta }{2}{∥D-P\hat{M}P^{T}∥}_{F}^{2},\\ & =\arg\min_{D}{∥K-D∥}_{1}+\frac{\beta }{2}{∥D-P\hat{M}P^{T}+\frac{\Lambda_{1}}{\beta }∥}_{F}^{2}, \end{array}$$

and the shrinkage (soft-thresholding) operator [22] is used to derive the solution:

(14) $$D_{+}\leftarrow K-S\left(K-P\hat{M}P^{T}+\frac{\Lambda_{1}}{\beta },\frac{1}{\beta }\right),$$

where S(x,τ)=sign(x)·max(|x|−τ,0) for a variable *x*.

To update *P*, the optimization problem is reduced as follows:

(15) $$\begin{array}{cc} P_{+} & =\arg\min_{P}\mathrm{tr}\left(\Lambda_{1}^{T}\left(D-P\hat{M}P^{T}\right)\right)+\frac{\beta }{2}{∥D-P\hat{M}P^{T}∥}_{F}^{2},\\ & =\arg\min_{P}\frac{\beta }{2}{∥D+\frac{\Lambda_{1}}{\beta }-P\hat{M}P^{T}∥}_{F}^{2}, \end{array}$$

subject to $P^{T}P=I_{r}$. The problem in Equation (15) is an orthogonality constrained least square problem. Let $R=D+\frac{\Lambda_{1}}{\beta }$ and $L=P\hat{M}$, then *L* is obtained by $L=R{\left(P^{T}\right)}^{+}=R{\left(P^{T}\right)}^{T}=RP$, where ${\left(P^{T}\right)}^{+}$ denotes the pseudo-inverse of $P^{T}$. Therefore, from [29], the orthogonal matrix *P* is computed by the QR factorization over *L*.

The optimization variable $\hat{M}$ is updated by solving the following equation:

(16) $${\hat{M}}_{+}=\arg\min_{\hat{M}}\mathrm{tr}\left(\Lambda_{1}^{T}\left(D-P\hat{M}P^{T}\right)\right)+\mathrm{tr}\left(\Lambda_{2}^{T}\left(\hat{M}-M\right)\right)+\frac{\beta }{2}\left(∥D-P\hat{M}P^{T}{∥}_{F}^{2}+{∥\hat{M}-M∥}_{F}^{2}\right),$$

and its closed-form solution is

(17) $${\hat{M}}_{+}=\frac{1}{2}\left(P^{T}DP+\frac{1}{\beta }P^{T}\Lambda_{1}P+M-\frac{1}{\beta }\Lambda_{2}\right).$$

Lastly, the Lagrange multipliers $\Lambda_{1}$ and $\Lambda_{2}$ are updated as follows:

(18) $$\begin{array}{cc} \Lambda_{1} & \leftarrow \Lambda_{1}+\beta \left(D-P\hat{M}P^{T}\right),\\ \Lambda_{2} & \leftarrow \Lambda_{2}+\beta \left(\hat{M}-M\right). \end{array}$$

The proposed structured kernel subspace learning method is summarized in Algorithm 1, which we call FactSPSD. In the algorithm, we compute the outputs by the scaling factor because a normalized observation is assumed in the proposed method. All optimization variables are set to have a value of zero for all experiments because initial values little change the final performance. The parameters of the algorithm are set to $\lambda =10^{-3}$, $\beta =10^{-5}$, and ρ=2. We set the number of inner iterations in lines 5–10 to 10 due to the empirical observation of convergence to a local solution. The convergence criterion described in line 13 in Algorithm 1 is chosen as

(19) $$∥D-P\hat{M}P^{T}{∥}_{1}<\epsilon \;\;\mathrm{or}\;\;{∥\hat{M}-M∥}_{1}<\epsilon ,$$

and ϵ is set to $10^{-5}$ for all our experiments. Note that it is difficult to specify the convergence to a local optimal solution rigorously due to the nonconvex and complicated problem. However, we empirically found that our algorithm converges to a stationary point within 30 iterations of the outer loop.
**Algorithm 1** FactSPSD (K,r,λ,β,ρ)1:Input: $K\in R^{n\times n}$, rank *r*, λ, β, and ρ2:Output: $P\in R^{n\times r}$ and $M\in R^{r\times r}$3:Initialization: $M=P=D=\hat{M}=0$ and $\beta_{max}=10^{10}$4:**while** not converged **do**5: **while** not converged **do**6:   Update *M* by Equation (12)7:   Update P←QR(RP) where $R=D+\frac{\Lambda_{1}}{\beta }$8:   Update $\hat{M}$ by Equation (17)9:   Update *D* by Equation (14)10: **end while**11: Update the Lagrange multipliers $\Lambda_{1}$ and $\Lambda_{2}$ by Equation (18)12: Update $\beta =\min(\rho \beta ,\beta_{max})$13: Check the convergence condition14:**end while**

Based on the structured low-rank approximation of a kernel matrix, we can derive a robust motion model using Gaussian process regression, as shown in Algorithm 2. The algorithm is named FactGP${}_{M}$ since it is based on factorization-based approach for Gaussian process regression. In Algorithm 2, standard PCA is performed to *L* in line 8 to remove the inverse operation, as in Equation (5), reducing the computational complexity from $O(n^{3})$ to $O(rn^{2})$. After training with the computed kernel matrix, we compute a new output using the trained components in the test phase.

**Algorithm 2** FactGP${}_{M}$1:Input: X,y, rank *r*, and $x_{*}$2:Output: ${\bar{y}}_{*}$3:// Training4: Compute $\Lambda =K+\sigma_{w}^{2}I$5: Perform kernel subspace learning:6:  [P,M] = FactSPSD(Λ,r,λ,β,ρ)7: $L\leftarrow PMP^{T}$8: Compute *R* and Σ by performing PCA to *L*9:// Testing10: Compute $k_{*}=k\left(x_{*},X\right)$11: Compute ${\bar{y}}_{*}$ by Equation (5)

## 4. Proposed Method: FactGP${}_{C}$

In this section, we propose an efficient and robust motion controller based on structured kernel subspace learning to avoid dynamic obstacles by assuming that there can be natural noises and inconsistent controls in the training set. The proposed motion controller utilizes both low-rank approximation for a set of controls and the proposed motion model.

### 4.1. Gaussian Process Motion Controller

It is usually required for a navigation algorithm to know trajectories of moving obstacles in the global frame of reference, whereas humans have an ability to navigate through a complex and crowded environment using local information collected from the egocentric view. The idea how a human reacts to a crowded environment is realized by Choi et al. [13] where they implemented a human-like navigation algorithm using the egocentric view of a robot which captures moving humans under a dynamic environment.

A mapping function F:T→U assigns a trajectory in $T\subset R^{2m}$, where *m* is the length of a trajectory in 2D, to a control input in $U\subset R^{2}$. In [13], a Gaussian process motion controller (GP-MC) is developed to find this mapping by assuming that a small variation in the trajectory space makes a small change in the control space, which can be seen as a continuous function. The covariance function for a GP-MC can be computed as follows:

(20) $$\mathrm{cov}\left(\tau_{i},\tau_{j}\right)=k_{u}\left(\tau_{i},\tau_{j}\right)+\sigma_{w}^{2}\delta_{ij},$$

where $\tau_{i}\in R^{2m}$ is the *i*th trajectory in the training set, which has *m* positions, and $\theta =\{\sigma_{f}^{2},\sigma_{x_{1}}^{2},\cdots ,\sigma_{x_{m}}^{2},\sigma_{w}^{2}\}$ are hyperparameters of a Gaussian process. When a new trajectory $\tau_{*}$ comes in, a motion control ${\bar{u}}_{*}$ for a robot can be computed using the GP-MC as follows:

(21) $${\bar{u}}_{*}=k_{u}{\left(\tau_{*},\tau_{tr}\right)}^{T}\Lambda_{u}y_{u},$$

where $\tau_{tr}=\left\{\tau_{1},\tau_{2},\cdots ,\tau_{N_{t}}\right\}$, $N_{t}$ is the number of trajectories in the training set, $\Lambda_{u}={\left(k\left(\tau_{tr},\tau_{tr}\right)+\sigma_{w}^{2}I\right)}^{-1}$, and $y_{u}$ is a vector of control outputs (directional and angular velocities) in the training set.

The GP-MC based motion control system does not assume external tracking systems. Instead, it uses the relative position information of pedestrians, which is coming from its egocentric sensor, as an input to the motion controller. To detect positions of pedestrians, a nearest-neighbor filter is used to assign a detection to known trajectories but a more sophisticated algorithm, such as multi-target tracking with data association [30], can be applied. When nearby trajectory is not detected in the field of view for an observed position, a new trajectory is formed.

### 4.2. FactGP${}_{C}$

A GP-MC is learned using a collected training set of pedestrian trajectories and corresponding control inputs to a robot. The training set usually contains a large amount of controls to represent a variety of controls like humans. To generate an effective training set for a GP-MC, a simulator, whose objective is to find an optimal shortest path to the goal point without collision, can be used to collect a diverse set of pedestrian trajectories and control inputs by densely sampling different initial positions, velocities, and accelerations of moving obstacles. Note that we assume that the dynamics of moving obstacles in a simulation follow the data of pedestrian behavior collected in a laboratory setting equipped with a Vicon motion capture system, and the behavior can be modeled by a Gaussian process. Thus, the proposed motion model can learn the behavior of pedestrians better than standard AR-GP. However, it is difficult to use a large amount of training data when we execute the GP-MC in the test phase because a larger training set requires more memory and computation time, making the algorithm unsuitable for real-time operations.

One can use random sampling to collect a subset of training data, but it does not preserve the diversity. To reduce the number of training samples while maintaining the diversity of training examples, a determinantal point process (DPP) [31] can be used to select an effective and diverse subset of training data. A DPP has been recently proposed to solve subset selection problems [31]. However, there can exist natural noises and inconsistent controls when trajectory-control pairs are collected from experiments or simulations. In addition, collected trajectories are also vulnerable to noises and outliers due to errors in sensors and detectors. These noises make the collected examples inconsistent and it can lead to unwanted situation when we execute a robot in a real environment.

To eliminate the effects of noisy or outlying training examples, we apply the proposed structured kernel subspace learning method to the GP-MC and propose a robust motion controller based on the approximated kernel matrix using the Gaussian process regression framework. The basic idea is similar to the proposed motion model in Section 3 in that it reduces the bad effects of unwanted measurements or noises. The motion controller is computed by approximating a kernel matrix $\Lambda_{u}$ as follows:

(22) $${\bar{u}}_{*}=k_{u}{\left(\tau_{*},\tau_{tr}\right)}^{T}\Lambda_{u}y_{u}\approx k_{u}{\left(\tau_{*},\tau_{tr}\right)}^{T}{\hat{\Lambda }}_{u}y_{u},$$

where ${\hat{\Lambda }}_{u}$ is computed by the structured kernel subspace learning algorithm described in Algorithm 1. The proposed motion controller based on structured kernel subspace learning is summarized in Algorithm 3. In the algorithm, the training phase is similar to the training phase of Algorithm 2. However, it is still a difficult task for a robot to react itself using a controller when a dynamic obstacle approaches to the robot rapidly. Hence, we predict the future position of a dynamic obstacle using the proposed motion model and the predicted position is combined with the recent positions of the obstacle which are fed into the motion controller to reduce collisions. In addition, the predicted positions are also used by the motion controller when dynamic obstacles disappear from the field of view of a robot for safer navigation.

**Algorithm 3** FactGP${}_{C}$1:Input: $\theta_{u},\tau_{1:N_{obs}},\tau_{tr}$, y, $y_{u}$, rank *r*, and $x_{robot}$2:Output: ${\bar{u}}_{robot}$3:// Training4: Compute $\Lambda_{u}=k_{u}\left(\tau_{tr},\tau_{tr}\right)+\sigma_{w}^{2}I$5: $\left[P_{u},M_{u}\right]$ = FactSPSD($\Lambda_{u},r,\lambda ,\beta ,\rho$)6: $L_{u}\leftarrow P_{u}M_{u}P_{u}^{T}$7: Compute $R_{u}$ and $\Sigma_{u}$ by performing PCA to $L_{u}$8: ${\hat{\Lambda }}_{u}={\tilde{R}}_{u}{\tilde{R}}_{u}^{T}$ using Equation (2)9: $\Gamma_{u}={\hat{\Lambda }}_{u}y_{u}$10:// Testing11: Compute $k_{u}^{*}=k_{u}\left(\tau_{new},\tau_{tr}\right)$12: Compute ${\bar{u}}_{robot}=k_{u}^{*}{\hat{\Lambda }}_{u}y_{u}$

## 5. Experimental Results

We evaluate the performance of the proposed methods (FactGP${}_{M}$ and FactGP${}_{C}$) in multiple tasks with various datasets and compare with other popular algorithms for Gaussian process regression (SPGP (available at http://www.gatsby.ucl.ac.uk/~snelson/) [32], PITC [33], GPLasso (available at https://www.cs.purdue.edu/homes/alanqi/softwares/softwares.htm) [25], and PCGP-$l_{1}$ [23]) along with the standard GP. For the motion control problem, we used the *k*-DPP [31] algorithm to select diverse trajectory-control pairs as a training set from collected examples. In our experiments, we used the radial basis kernel function for all AR-GP approaches and hyperparameters used in AR-GP are learned by a conjugate gradient method [17]. The root mean squared error (RMSE) is used as an accuracy measure for prediction and regression problems. We conducted all simulations using an MATLAB environment on a computer with 16 GB RAM and a 3.4 GHz quad-core CPU.

### 5.1. Regression Problems

First, we tested the proposed structured low-rank matrix approximation method on a synthetic regression problem. We compared FactGP${}_{M}$ to a sparse GP (PITC [33]) and the full GP [17] to observe how different methods perform in the presence of corruptions.

**Figure 3:**
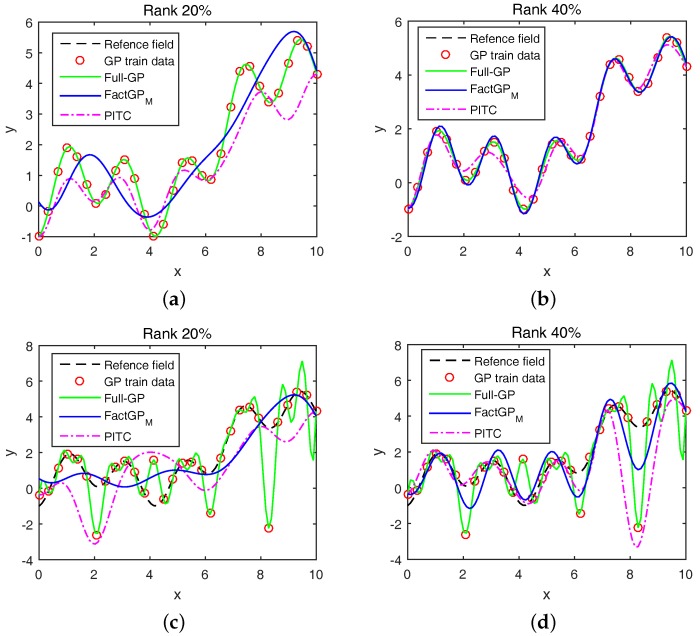
Simulation results on a synthetic example with and without outliers. FactGP${}_{M}$ and PITC use kernel matrices whose ranks are either 20% or 40% of the size of the original kernel matrix: (**a**) No outliers with 20% low-rank; (**b**) no outliers with 40% low-rank; (**c**) 20% outliers with 20% low-rank; and (**c**) 20% outliers with 40% low-rank.

Figure 3 shows the regression results in the case where no outliers and 20% outliers exist. We also compared the low-rank approximation methods, FactGP${}_{M}$ and PITC, at two different ranks (while PITC is a sparse GPR method, we treat it as a low-rank approximation method since the rank can be considered as a generalization of sparsity for two-dimensional data) (20% and 40% of the size of the kernel matrix). When there are no outliers, the full GP exactly fits the reference field but FactGP${}_{M}$ and PITC show smooth lines with 20% low-rank components as shown in Figure 3a. However, the low-rank approximation methods try to fit the reference field with the larger rank (40%), as shown in Figure 3b. However, PITC still does not fit the reference very wellm as it misses some samples. Our method gives competitive results compared to other methods in the regression problem. When we add outliers to randomly selected 20% of data mas shown in Figure 3c,d, the full GP and PITC are significantly affected by outliers, showing large fluctuations. However, FactGP${}_{M}$ is less affected by outliers, showing its robustness against outliers.

**Figure 4:**
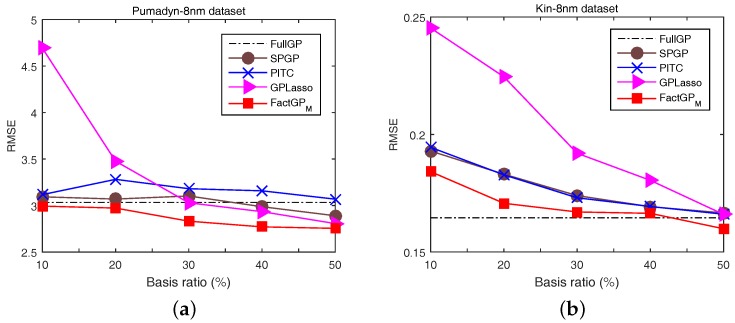
Errors of the proposed FactGP${}_{M}$ and compared methods under various basis ratios for two regression problems: (**a**) Pumadyn; and (**b**) Kin. Note that the figure are borrowed from our preliminary work [24] (reproduced with permission from Eunwoo Kim, Sungjoon Choi, Songhwai Oh, Structured Low-Rank Matrix Approximation in Gaussian Process Regression for Autonomous Robot Navigation; published by IEEE, 2015).

We also tested the proposed method using real-world datasets, Pumadyn-8nm (Pumadyn) and Kin-8nm (Kin) (available at http://www.cs.toronto.edu/~delve/methods/mars3.6-bag-1/mars3.6-bag-1.html— both datasets are frequently used to measure the performance of different Gaussian process regression methods) [25] and randomly collected 1000 train and 800 test samples for each dataset. We modified the datasets by adding 30% outliers randomly selected from [−25, 25] to verify the robustness of the proposed algorithm, whereas original data values are in the range of [−2, 2]. Figure 4 shows the simulation results of our proposal with other sparse Gaussain process regression (GRP) methods, SPGP [32], PITC [33], and GPLasso [25], for various basis ratios from 10% to 50%. The proposed method performs better than other compared methods regardless of the basis ratios as shown in the figure. Moreover, it performs better than the full GP which gives lower error than sparse GPR methods for cases when the basis ratio is small. Figure 4b also shows the excellence of the proposed method compared to other sparse GPR methods.

### 5.2. Motion Prediction of Human Trajectories

**Figure 5:**
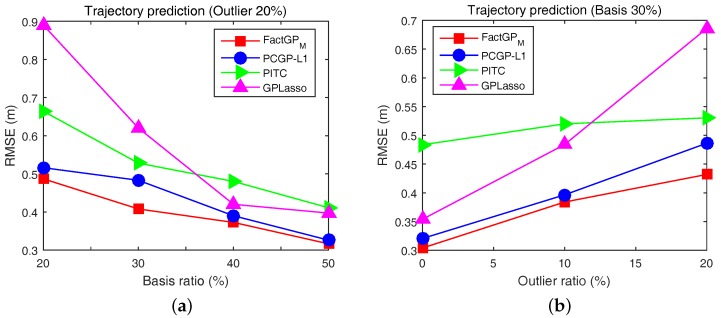
Motion prediction results based on human trajectories with respect to: (**a**) various basis ratios in the existence of 20% outliers; and (**b**) various outliers while 30% basis ratio is fixed.

**Figure 6:**
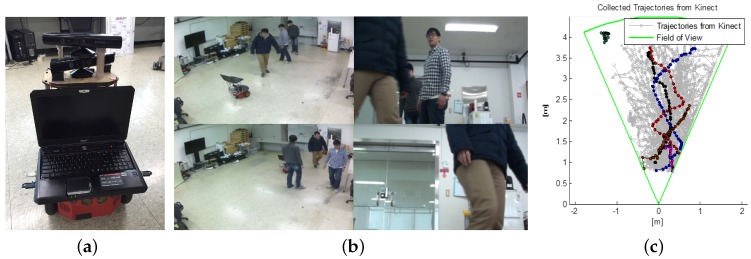
(**a**) A mobile robot equipped with two Kinect cameras; (**b**) snapshots from an experiment in a human–robot environment, where the first column is a third-person view, while second column is the egocentric view of a robot; and (**c**) collected trajectories [23] (reproduced with permission from Eunwoo Kim, Sungjoon Choi, Songhwai Oh, A Robust Autoregressive Gaussian Process Motion Model Using *l*_1_-Norm Based Low-Rank Kernel Matrix Approximation; published by IEEE 2014). For better visualization, we represent some trajectories in color.

For motion prediction, we collected human trajectories, where we use three past positions in absolute coordinates, using the Pioneer 3 DX robot platform equipped with a Microsoft Kinect camera (For the motion prediction experiment, we collected human trajectories using one Microsoft Kinect camera and the experimental results are shown in Figure 5. However, for other experiments, we used two Kinect cameras to increase the field of view of the robot), as shown in Figure 6a. All performed algorithms in this problem were written in MATLAB with the mex-compiled ARIA package (available at http://robots.mobilerobots.com/wiki/ARIA) and conducted on a computer with a 2.5 GHz quad-core CPU and 8 GB RAM. The position of a moving object is detected by the human skeleton tracking API in Kinect.

Experiments to estimate the future position of an individual moving person were conducted in our laboratory, where the future trajectories are modeled by AR-GP [13]. Let $D_{t}\in R^{2}$ be the position of a pedestrian at time *t*. The current velocity, $\Delta D_{t}=D_{t}-D_{t-1}$, is modeled in AR-GP as follows [13], with an appropriate time scaling:

(23) $$\Delta D_{t}=f\left(D_{t-1},D_{t-2},\cdots ,D_{t-p}\right)∼GP_{f}\left(D_{t-1},D_{t-2},\cdots ,D_{t-p}\right).$$

Hence, the AR-GP motion model can find the position of a pedestrian at time *t* based on *p* recent positions of the pedestrian with the nonlinear autoregressive model.

Figure 6b shows snapshots from the third-person (left) view and the egocentric view (right) from a robot. Some of the collected trajectories of pedestrians from the filed of view of a robot are illustrated in Figure 6c. We made a training set from the trajectories, where the number of positions in a trajectory was set to ten. When a trajectory has many detected positions, we uniformly collected ten positions. From a trajectory which has *n* positions, we obtain n−p+1 input samples where *p* is the order of an autoregressive motion model, i.e., the number of past positions. One can model it as a Hankel matrix by shifting one point in a trajectory.

**Figure 7:**
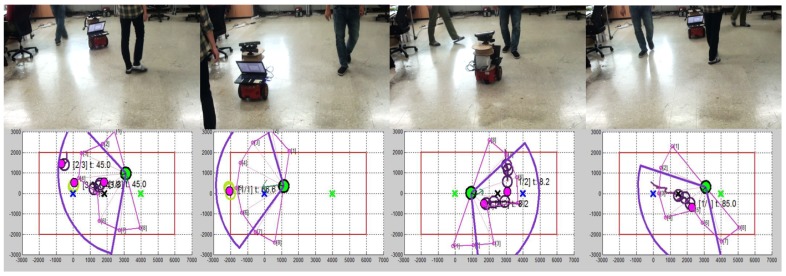
Motion prediction experiments using the proposed motion model, FactGP${}_{M}$ [24] (reproduced with permission from Eunwoo Kim, Sungjoon Choi, Songhwai Oh, Structured Low-Rank Matrix Approximation in Gaussian Process Regression for Autonomous Robot Navigation; published by IEEE, 2015). A pink circle is the prediction made by FactGP${}_{M}$ given past pedestrian positions (purple or yellow-green circles). The violet fan-shaped region is the field of view of two Kinect sensors and the pink fan-shaped region shows sensing responses from sonar sensors of the Pioneer robot. Each column consists of a photo taken by a camera and the internal state of the robot. Best viewed in color.

The proposed method was compared with existing approaches including PCGP-$l_{1}$ [23], GPLasso [25], and PITC [33]. We set the order of autoregressive to p=3 to divide the collected trajectories into train and test sets. We conducted experiments for two scenarios: (1) under various basis (rank) conditions while fixing an outlier ratio; and (2) under various outlier ratios while fixing the number of bases (rank). Outliers were randomly added from [−10, 10] to the trajectories, whereas the original trajectories are in the range of [−5, 5]. Computed prediction errors by tested algorithms are shown in Figure 5, where our approach FactGP${}_{M}$ gives the best performance in all cases. Another $l_{1}$-norm based approach, PCGP-$l_{1}$ shows the second best results. Here, we can interpret that satisfying the positive semi-definite (PSD) property gives stable solutions whose results perform better than the method without the PSD guarantee (such as PCGP-$l_{1}$). Figure 5b shows the results using RMSE for a fixed rank (r/n×100=30%) with outliers. Similar to the previous experiments, the proposed approach shows the excellence performance under various outlier conditions. We also conducted a motion prediction experiment in a real environment with moving humans. Here, a robot with a proper sensing range observes moving objects when the objects are detected from the camera sensors and predicts the next positions of them. Then, given the past and future positions the robot determines its control to avoid collision with the moving obstacles while pursuing the shortest path to the goal. Collected snapshots from the experiment in our laboratory is shown in Figure 7, where we used two Microsoft Kinect cameras whose field of view is around $110^{\circ }$. The robot performed the nearly exact prediction of the future positions of pedestrians in real-time (around 10 ms for a prediction in our experimental environment).

### 5.3. Motion Control

To collect an enough number of training samples for FactGP${}_{C}$, we collected training samples, i.e., trajectory-control pairs, using a computer simulator, as done in [13]. A total of 8845 trajectory-control pairs are collected from simulation. From the collected samples, we selected 3000 samples using DPP [31] and another 3000 samples were selected randomly. We compared the proposed motion controller with the Gaussian process motion controller (GP-MC) [13], which is based on the standard full GP, vector field histogram (VFH) [18], and reactive planner (Reactive) [9]. In addition, we compared with AR-VFH, which is based on VFH but using future positions predicted by AR-GP, to verify whether the motion prediction is helpful to VFH for autonomous navigation. The proposed method is applied at three different rank levels: 10%, 20%, and 40% of the full rank. Since there can be more than one obstacle, a robot first predicts future positions of multiple obstacles and then uses the closest future point with its corresponding recent positions for a motion control.

In simulation, the number of dynamic obstacles is varied from one to six. Considering the fact that the sensing range of the robot is about 5 m, we are considering crowded situations. When the distance between the center of a robot and the center of an obstacle is less than 500 mm, a collision is declared. The collision rate is computed as follows:

(24) $$\mathrm{Collision}\;\mathrm{rate}\left(\%\right)=\frac{\mathrm{number}\;\mathrm{of}\;\mathrm{collided}\;\mathrm{objects}}{\mathrm{total}\;\mathrm{number}\;\mathrm{of}\;\mathrm{objects}}\times 100.$$

Table 1 shows the average collision rate (ACR) and the minimum distance (MinD) (MinD is the most minimal distance among distances to moving objects for all time steps until a robot reaches the goal from the starting point) to obstacles of different motion control algorithms (note that MinD and navigation time are computed using cases with no collisions for a fair comparison). The average collision rate for each case is computed from 30 independent trials. Inthe table, FactGP${}_{C}$ with 20% basis vectors gives the best performance, whereas GP-MC shows a higher collision rate than the proposed method on average. Note that a 20% compression rate can perform better than 40% under noisy scenarios since the impact of unwanted noises can be weaker at a higher compression rate. In the experiment, GP-MC sometimes makes an excessive detour when an obstacle approaches or makes a brief stop when obstacles disappear from the field of view of a robot, which can lead to a collision with an obstacle. AR-VFH reduces the collision rate by predicting future positions of moving obstacles compared to VFH, showing the benefit of predicting future positions of moving obstacles in dynamic environments. In terms of the minimum distance to obstacles, all methods except VFH show similar distances. Reactive shows performance better than VFH-based methods, but Reactive is still poor when compared with GP-MC or FactGP${}_{C}$. VFH gives the worst performance with respect to both collision rates and MinD in all cases.

**Figure 8:**
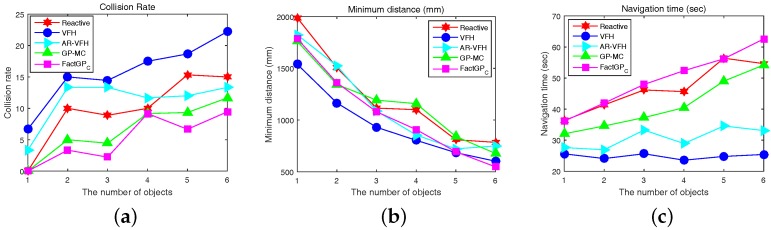
A comparison of motion controllers at different numbers of obstacles with respect to: (**a**) collision rate (%); (**b**) minimum distance (mm); and (**c**) navigation time (s). FactGP${}_{C}$ (20) and GP-MC are trained using 3000 samples selected by DPP.

**Figure 9:**
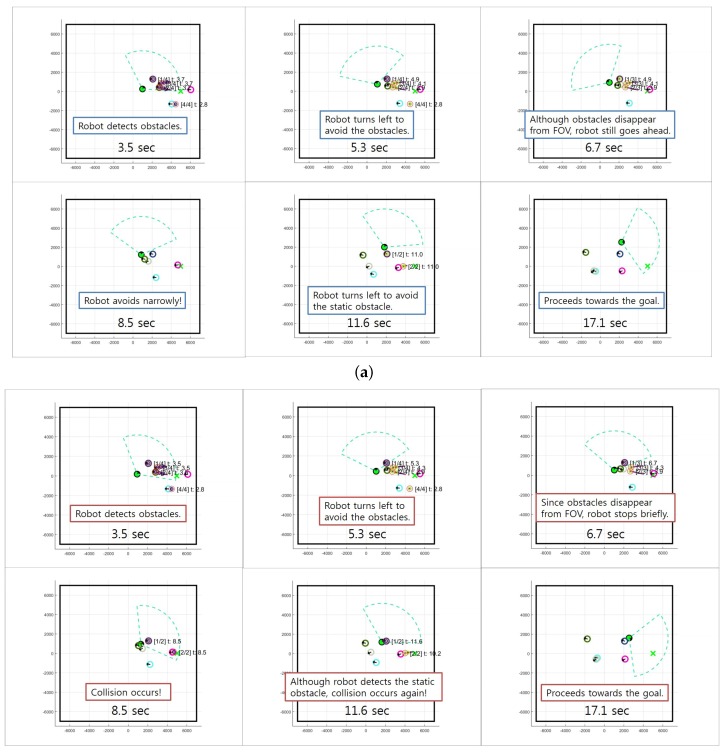
Snapshots from a motion control simulation with five dynamic obstacles using: (**a**) FactGP${}_{C}$; and (**b**) GP-MC.

We also compared the proposed motion controller with GP-MC using 3000 training samples selected by DPP. We used FactGP${}_{C}$ with 20% rank in this experiment because its performance is better than the others, as shown in the previous experiment. The experimental results of all methods with respect to the collision rate, minimum distance, and navigation time over 30 independent trials are shown in Figure 8. In Figure 8a, FactGP${}_{C}$ shows the lowest collision rates in all cases. GP-MC shows the second best performance among the methods. When it comes to the minimum distance to obstacles, all methods show the similar trend as the case using the entire dataset, as shown in Figure 8b. VFH shows the lowest minimum distance to obstacles on average while Reactive gives the highest minimum distance on average. Average navigation times are shown in Figure 8c. FactGP${}_{C}$ requires the longest navigation time because it reacts quickly to incoming obstacles and actively avoids collisions. (for example, see Figure 9). VFH and AR-VFH require less navigation times than other methods but they give higher collision rates. Figure 9 shows some snapshots for a scenario with five moving obstacles using the proposed FactGP${}_{C}$ with 20% low-rank basis and GP-MC. In the scenario, a robot with green circle starts at (0, 0) and should arrive at the goal (5000, 0), while moving obstacles with different circles of different color move from around the goal toward left side with different velocity. When a moving object comes inside the field of view of the robot, the robot detects the trajectory of the moving object and predicts the next position. Then, the robot controls to avoid the nearest position among the predicted positions of multiple obstacles while pursuing the shortest route the to goal. Here, a robot controlled by FactGP${}_{C}$ avoids collisions with obstacles by reacting rapidly when moving obstacles disappear from the field of view of the robot, whereas a robot controlled by GP-MC collides with obstacles by making a brief stop (simulation times 5.3 s and 6.7 s). We can see that the proposed controller does not use redundant controls from the collected training set, which leads to make quick and safe controls.

**Figure 10:**
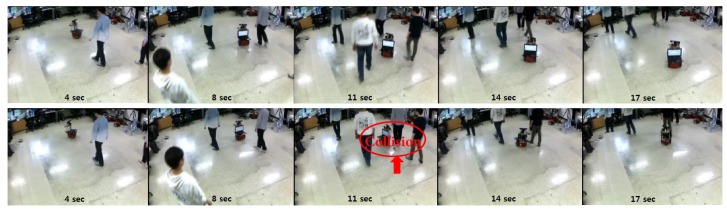
Snapshots from a motion control experiment in a laboratory with four moving pedestrians using: FactGP${}_{C}$ (**top**); and GP-MC (**bottom**) .

We applied the proposed motion controller in the same experimental setting to the motion prediction experiments with two Kinect cameras. The number of moving objects varies from one to four in the experiments to demonstrate the performance of the proposed algorithm under crowded environments. The proposed method is compared with GP-MC [13]. Figure 10 shows snapshots from the experiment with four pedestrians. The goal was to navigate to the pre-assigned goal region without collision. From the figure, a robot using the proposed controller navigated safely by actively avoiding incoming pedestrians, while the robot using GP-MC was not successful at avoiding the incoming pedestrian (at 11 s). The average collision rate with trials (%) is computed as

(25) $$\mathrm{Collision}\;\mathrm{rate}\;\mathrm{with}\;\mathrm{trials}\;\left(\%\right)=\frac{\mathrm{no}.\;\mathrm{trials}\;\mathrm{with}\;a\;\mathrm{collision}}{\mathrm{total}\;\mathrm{number}\;\mathrm{of}\;\mathrm{trials}}\times 100.$$

Table 2 shows the average collision rates of two methods at different numbers of obstacles from 10 independent trials in our laboratory. On average, GP-MC shows a collision rate of 25% while the proposed FactGP${}_{C}$ shows a collision rate of 10% for this experiment (Note that the collision rates shown in Table 2 are higher than the numbers reported in Table 1. This is due to the fact that the collision rate is computed differently, as shown in Equation (25). The collision rate in Equation (25) is computed in terms of the number of trials while the collision rate in Equation (24) is computed in terms of the number of objects. In addition, the moving speed of pedestrians and invalid detections of Kinect sensors are also contributing factors).

**Figure 11:**
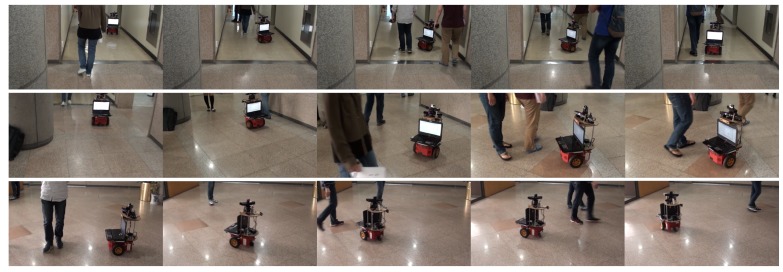
Snapshots from a real autonomous robot navigation experiment using the proposed motion model and motion controller in an L-shape lobby.

**Figure 12:**
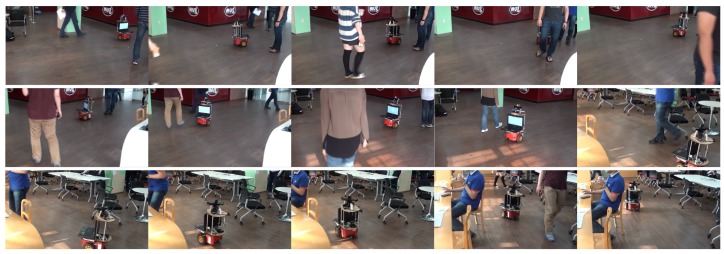
Snapshots from a real autonomous robot navigation experiment using the proposed motion model and motion controller in a school cafeteria.

The proposed motion model and motion controller are applied in other environments, including an L-shape lobby and a school cafeteria. The goal was to reach the goal region under a dynamic environment with many moving pedestrians. Figure 11 and Figure 12 show some snapshots from the experiments in an L-shape lobby and a crowded school cafeteria, respectively. In all experiments, the robot with the proposed motion model and controller successfully navigated without collisions by avoiding pedestrians and arrived at the goal region.

## 6. Conclusions

In this paper, we have proposed FactGP${}_{M}$ and FactGP${}_{C}$ for motion prediction and motion control problems, respectively, based on the proposed robust kernel matrix approximation method, FactSPSD. We have proposed a novel formulation by considering the limitations of existing approximation methods and solved it under the augmented Lagrangian framework. The approximation finds low-rank kernel subspace by minimizing a nuclear-norm regularized $l_{1}$-norm objective function. The proposed method has been applied to an extensive set of experiments including well-known regression problems and motion prediction and control problems under real-world environments using a mobile robot with Kinect cameras. In experiments, we have shown the efficiency and robustness of the proposal against unwanted outliers, measurement errors, and inconsistent controls in the training set compared to existing methods.

## Figures and Tables

**Table 1:** Average collision rate (%) and minimum distance (mm) of our FactGP${}_{C}$ at three different rank ratios (10%, 20%, and 40%) as well as GP-MC, VFH, AR-VFH, and Reactive when there are different numbers of moving obstacles.

		Ours (10)	Ours (20)	Ours (40)	GP-MC	VFH	AR-VFH	Reactive
1 object	ACR	0	0	0	3.33	6.67	3.33	0
	MinD	1969	1839	1708	1761	1539	1824	1988
2 object	ACR	6.67	3.33	3.33	5.0	15.0	13.33	10.0
	MinD	1422	1400	1300	1344	1165	1523	1506
3 object	ACR	6.67	6.67	8.89	8.89	14.44	13.33	8.89
	MinD	1062	1228	1254	1218	930.2	1087	1116
4 object	ACR	6.67	8.83	8.83	8.83	17.5	11.67	10.0
	MinD	839.4	951.7	1083	1144	804	855	1100
5 object	ACR	11.33	8.67	12.0	8.0	18.67	12.0	15.33
	MinD	836.2	785.9	1022	861	684.9	721.4	809.4
5 object	ACR	16.67	12.78	9.44	12.78	22.22	13.33	15.0
	MinD	928.9	757.7	1008	783.8	602	749.9	784.5
Average	ACR	8.01	6.63	7.00	7.72	15.75	11.16	9.87
	MinD	1126.3	1160.4	1229.2	1185.3	954.2	1126.7	1217.3

**Table 2:** Average collision rate with trials (%) of FactGP${}_{C}$ with 20% basis vectors and GP-MC.

Algorithm	#obs 1	#obs 2	#obs 3	#obs 4	Average
FactGP${}_{C}$	0%	0%	20%	20%	10%
GP-MC	0%	20%	30%	50%	25%
